# Phytochemical, antimicrobial, and antioxidant activities of different citrus juice concentrates

**DOI:** 10.1002/fsn3.268

**Published:** 2015-07-30

**Authors:** Ehigbai I. Oikeh, Ehimwenma S. Omoregie, Faith E. Oviasogie, Kelly Oriakhi

**Affiliations:** ^1^ Department of BiochemistryFaculty of Life SciencesUniversity of BeninPMB 1154Benin CityNigeria; ^2^ Department of MicrobiologyFaculty of Life SciencesUniversity of BeninPMB 1154Benin CityNigeria; ^3^ Department of Medical BiochemistryFaculty of Basic Medical SciencesUniversity of BeninPMB 1154Benin CityNigeria

**Keywords:** Antimiocrobial, antioxidant, citrus, juice concentrate

## Abstract

The search for new antimicrobial compounds is ongoing. Its importance cannot be overemphasized in an era of emerging resistant pathogenic organisms. This study therefore investigated the phytochemical composition and antioxidant and antimicrobial activities of different citrus juice concentrates. Fruit juices of *Citrus tangerine* (tangerine), *Citrus paradisi* (grape), *Citrus limon* (lemon), and *Citrus aurantifolia* (lime) were evaluated. Antimicrobial activities against five bacterial and three fungal strains were evaluated. The results revealed the presence of alkaloids, flavonoids, steroids, terpenoids, saponins, cardiac glycosides, and reducing sugars in all the juice concentrates. DPPH (1,1‐diphenyl‐2‐picrylhydrazyl) radical scavenging capacities varied with tangerine and grape juices having better scavenging capacities than lemon and lime juices. Grape juice was observed to have a significantly higher (*P* < 0.05) ferric‐reducing antioxidant potential (FRAP) value (364.2 ± 10.25 *μ*mol/L Fe(II)/g of the extract) than the reference antioxidant, ascorbic acid (312.88 ± 5.61 *μ*mol/L). Antimicrobial studies revealed differential antimicrobial activities against different microbial strains. Zones of inhibition ranging from 4 to 26 mm were observed for the antibacterial tests with 0–24 mm for antifungal test. Minimum inhibitory concentrations (MIC) and minimum bacteriostatic concentrations (MBC) for concentrates against bacterial strains ranged from 12.5 to 200 *μ*g/mL. Lemon and lime juice concentrates had lower MIC and MBC values with orange and tangerine having the highest values. Minimum fungicidal concentrations ranged from 50 to 200 *μ*g/mL. The results of this study suggest that these juice concentrates may have beneficial antimicrobial roles that can be exploited in controlling unwanted microbial growth.

## Introduction

Normal physiological processes in vivo result in the production of free radicals. Oxidative stress results when there is an insufficient capacity of the biological system to neutralize excess free radicals that have been produced. This may result in aging and disease conditions (Sahreen et al. 2014). Food and fruits are known to contain antioxidants that are linked to in vivo protection from oxidative stress (Jensen et al. 2008).

An estimated 100 million tons of citrus fruits are produced annually; thereby making the citrus family the largest contributor to the world's fruit production (Jwanny et al. 2012). Citrus is one of the largest plant species known, consisting of 40 species that are distributed around the world (Karimi et al. 2012). Among the most commonly consumed citrus fruits in Nigeria are tangerine, lime, lemon, and grape.

Citrus juices are consumed majorly because of their nutritional value and special flavor. Fruit juice consumption is beneficial for the maintenance of good health and prevention of diseases. The positive health benefits of juices have been ascribed in part to vitamin C (ascorbic acid), the major vitamin found in fruits and vegetables (Boudries et al. 2012; Rekha et al. 2012). Citrus fruits are also known to contain bioactive compounds such as phenolics, flavonoids, vitamins, and essential oils which are believed to be responsible for a range of protective health benefits including antioxidative, anti‐inflammatory, antitumor, and antimicrobial activities (Aruoma et al. 2012; Karimi et al. 2012).

The problem of resistance of microorganisms to antimicrobial drugs is one of the world's current challenges. On the other hand, plant‐based antimicrobials are attractive as they are often devoid of the many side effects associated with synthetic antimicrobials. The search for new antimicrobial compounds from natural sources is, thus, an ongoing one (Parekh et al. 2005).

This study was therefore aimed at evaluating the phytochemical composition and antioxidant and antimicrobial activities of different commonly available citrus juice concentrates in Nigeria.

## Materials and Methods

### Collection and identification of plant materials

Fresh fruits (tangerine, lemon, lime, and grape) were purchased from New Benin Market, Benin City in Edo State, Nigeria. The fruits were identified by a Botanist in the Department of Plant Biology and Biotechnology of the University of Benin, Benin City, Nigeria.

### Preparation of fruit juice concentrates

The fruits were rinsed thoroughly with distilled water and were cut into halves. The juice was extracted from the fruits using a juice extractor. The fruit juices were then lyophilized and the concentrates obtained were preserved at 4°C in airtight containers until subsequent use.

### Phytochemical screening

Phytochemical screening was carried out on the juice concentrates using established protocols as described by Trease and Evans (1989), Sofowora (1993), and Harborne (1998).

Stock solutions of each extract with a concentration of 10 mg extract/mL distilled water was prepared and used for the phytochemical screening.

### Estimation of antioxidant activity

#### Determination of DPPH radical scavenging activity

Radical scavenging activity of each concentrate against the 1, 1‐diphenyl‐1‐picryl‐hydrazyl radical (DPPH) was done by a slightly modified method by Brand‐Williams et al. (1995). Concentrations of each sample was prepared in methanol and used for the assay. Ascorbic acid was used as standard, and the same concentrations, as the test solution in methanol were prepared. Two milliliters of the prepared concentration was placed into test tubes and 0.5 mL of 1 mmol/L DPPH solution in methanol was added. The experiment was carried out in triplicates. The test tubes were incubated for 15 min at room temperature, and the absorbance was read at 517 nm. A blank solution containing the same amount of methanol and DPPH was prepared and the was absorbance read. Lower absorbance of the reaction mixture indicates higher free radical scavenging activity. The radical scavenging activity was calculated using the following formula:

$$DPPHradicalscavengingactivity\left(\%\right)=[\left(A_{0}-A_{1}\right)/\left(A_{0}\right)]\times 100,$$

where *A*_0_ was the absorbance of DPPH radical + methanol; *A*_1_ was the absorbance of DPPH radical + sample or standard. The 50% inhibitory concentration value (IC_50_) is indicated as the effective concentration of the sample that is required to scavenge 50% of the DPPH free radical.

### Ferric‐reducing antioxidant power assay

The Benzie and Strain (1996) protocol with slight modification was employed for this assay. Different concentrations (100–500 mg/mL) of the concentrates and the standard were serially diluted with distilled water. One milliliter of FRAP reagent (200 mL of 300 mmol/L sodium acetate buffer at pH 3.6, 20 mL of 10.0 mmol/L 2,4,6‐tripyridyl ‐s‐ triazine (TPTZ) solution, 20 mL of 20.0 mmol/L FeCl_3_.6H_2_O solution, and 24 mL of distilled water) was then added to each test tube. The resulting mixture was vigorously shaken and then incubated at 37°C for 4 min and the increase in absorbance at 593 nm was measured and compared with the standard ascorbic acid.

### Antimicrobial assay

#### Test microorganisms

Eight microorganisms were used in this study, consisting of five bacterial strains and three fungal strains. Two were gram positive (*Staphylococcus aureus* and *Enterococcus faecalis*), while the other three were gram negative (*Pseudomonas aeruginosa*,* Escherichia coli*, and *Salmonella* spp.). The three fungal strains used are *Candida albicans*,* Aspergillus niger*, and *Penicillum* spp. All microorganisms were obtained from Lahor Research and Diagnostic Laboratories, Benin City, Nigeria.

### Antimicrobial susceptibility assay

This was carried out using the protocol described by Owoseni and Ajayi (2010). Test organisms were subcultured onto fresh suitable broth medium. Broth cultures were then incubated at 37°C till the turbidity of 0.5 McFarland's standard was obtained. Mueller–Hinton agar was used as bacterial medium and Sabouraud agar as fungal medium. All were incubated appropriately as specified for each test organism. The turbidity of the actively growing broth culture was then adjusted with sterile saline to obtain 0.5 McFarland's standard turbidity. This was used to flood the surface of solid Mueller–Hinton agar plates and then drained dry. Wells of 5 mm in diameter and about 2 cm apart were punched in the culture media with sterile cork borer. The extracts were thereafter used to fill the boreholes. Each plate was kept in the refrigerator at 4°C for 1 h before incubating at 37°C for 24 h (bacteria) and 72 h (fungi). Zones of inhibition around the wells, measured in millimeters, were used as positive bioactivity.

### Minimum inhibitory concentration

The organisms that showed susceptibility to different solvent extracts were introduced into the broths containing different concentrations of each extract (serial dilutions of the extracts corresponding to 200, 100, 50, 25, and 12.5 *μ*g/mL). The tubes were then incubated for 24 h at 37°C. The minimum inhibitory concentrations (MIC) was taken as the lowest concentration of the extracts that did not permit any visible growth (Owoseni and Ajayi 2010).

### Minimum bactericidal concentration and minimum fungicidal concentration

The tubes that showed no turbidity in the MIC test were taken and a loop‐full from each tube was streaked on Mueller–Hinton agar. The plates were incubated for 24 h at 37°C and the absence of growth was observed. The concentration of the extracts that showed no growth was recorded as the minimum bactericidal concentration (MBC)/minimum fungicidal concentration (MFC) (CLSI 2008a,b, 2012).

### Statistical analysis

The data were expressed as mean ± SEM of three replicates. The data were subjected to one‐way analysis of variance (ANOVA), and differences between means were determined by Duncan's multiple range test using the Statistical Analysis System (SPSS Statistics 17.0, SPSS Inc. Chicago, Illinois, USA) where applicable. *P* ≤ 0.05 were regarded as significant.

## Results and Discussion

Phytochemicals are non‐nutritive plant chemicals possessing varying degrees of disease‐preventive properties. They are invaluable sources of raw materials for both traditional and orthodox medicine (Oikeh et al. 2013). In this study, the phytochemical composition of the citrus juice concentrates revealed the presence of alkaloids, flavonoids, steroids, terpenoids, saponins, cardiac glycosides, and reducing sugars (Table 1). However, the abundance of these phytochemicals varied from juice to juice. These results agree in part with the findings of Rauf et al. (2014) who reported the presence of reducing sugars, phenols, flavonoids, and terpenoids in lemon and lime juice. However, they did not detect the presence of alkaloids, saponins, and glycosides, while steroids was absent in lemon. These differences may be due to differences in species and geographical location.

**Table 1:** Phytochemical composition of some citrus fruit juice concentrates

	Tangerine	Grape	Lemon	Lime
Alkaloids	++	+	+	+
Phenols	+	+++	+	+
Flavonoids	+	+++	+	+
Steroids	++	+	+	+
Terpenoids	++	+	+	+
Reducing sugar	+	+	+	++
Saponins	++	++	+	+
Cardiac glycosides	+	+	+	++

+, slightly detected; ++, moderate amount; +++, high amounts.

Generally, phytochemicals are known to confer certain health benefits such as anti‐inflammatory, antimicrobial, antihypertensive, and antidiabetic effects (Ayoola et al. 2008; Oikeh et al. 2013).

The presence of flavonoids, alkaloids, steroids, terpenoids, saponins, cardiac glycosides, and reducing sugars in all the juice concentrates studied corroborates the assertion of Mathew et al. (2012) that citrus fruits are rich sources of phytochemicals.

The DPPH radical scavenging assay is a commonly used tool for accessing the antioxidant capacity of plant materials because of the relative cheap cost and speed of completion.

The results from this study showed increase in the radical scavenging activities of all extracts as the concentration increased from 0.5 to 1.0 mg/mL (Table 2). The standard antioxidant, ascorbic acid had significantly higher (*P* < 0.05) percentage inhibitions of the DPPH radical than all the juice concentrates at both concentrations studied (40.10 ± 0.02% and 70.20 ± 0.20% at concentrations of 0.5 and 1.0 mg/mL of extract). Tangerine had the highest DPPH radical scavenging activity of the four studied juice concentrates (17.08 ± 0.60% and 26.45 ± 0.15% inhibition). A comparison of the DPPH radical scavenging activities of the lemon and lime juices corroborates the finding of Rauf et al. (2014) who observed better percentage inhibition of the DPPH radical in lime compared to lemon at all concentrations studied.

**Table 2:** Percentage inhibition of the 1,1‐diphenyl‐2‐picrylhydrazyl radical scavenging activity of some citrus fruit juice concentrates

Sample	% inhibition (mg/mL)	
	0.5	1.0
Ascorbic acid	40.10 ± 0.02	70.20 ± 0.20
Tangerine juice concentrate	17.80 ± 0.60*	26.45 ± 0.15*
Grape juice concentrate	2.60 ± 0.30*	24.0 ± 0.10*
Lemon juice concentrate	2.60 ± 0.20*	5.25 ± 0.40*
Lime juice concentrate	2.85 ± 0.20*	6.25 ± 0.06*

Values are expressed as mean *± *SEM. *n = *3*/*group.

Values in a column with (*) are significantly different (*P* < 0.05) from the reference antioxidant.

The results also revealed that the juice concentrates are not as effective as DPPH radical scavengers when compared to ascorbic acid. At the concentrations studied, ascorbic acid has a significantly higher inhibition of the DPPH radical compared to the juice concentrates.

The FRAP of the citrus juice concentrates revealed that the tangerine, lemon, and lime juice concentrates had significantly lower FRAP values (179.75 ± 4.25, 122.75 ± 3.25, and 173.25 ± 0.25 *μ*mol/L Fe(II)/g of the extract, respectively) compared to the reference antioxidant (312.88 ± 5.61 *μ*mol/L Fe(II)/g of the extract). Grape juice concentrate had a higher FRAP value (364.2 ± 10.25 *μ*mol/L Fe(II)/g of the extract) than that of ascorbic acid (Table 3). This difference was however not significant (*P* > 0.05). The results suggest that grape juice has a good reductive power and may thus possess significant antioxidant activity.

**Table 3:** Ferric‐reducing antioxidant potential (FRAP) of some citrus fruit juice concentrates

Plant extract	FRAP value (*μ*mol/L Fe(II)/g) of the extract
Ascorbic acid	312.88 ± 5.61
Tangerine juice concentrate	179.75 ± 4.25*
Grape juice concentrate	364.2 ± 10.25*
Lemon juice concentrate	122.75 ± 3.25*
Lime juice concentrate	173.25 ± 0.25*

Values are expressed as mean *± *SEM. *n = *3*/*group.

Values with (*) are significantly different (*P* < 0.05) from the reference antioxidant.

The antibacterial susceptibility test of the juice concentrates against some gram‐positive and ‐negative bacterial strains are shown in Table 4. The observed zones of inhibition varied from one organism to another and from one citrus juice concentrate to another,

**Table 4:** Antibacterial activities of some citrus juice concentrates (200 *μ*g/mL) against some bacterial strains tested by disc diffusion assay

	Zone of inhibition (mm)				
	Gram positive		Gram negative		
	*Staphylococcus aureus*	*Enterococcus faecalis*	*Pseudomonas aeruginosa*	*Escherichia coli*	*Salmonella* spp.
Tangerine	18	26	25	18	6
Grape	10	8	8	4	6
Lemon	20	10	18	12	10
Lime	20	14	16	8	10

The gram‐positive strains showed higher susceptibility values than the gram‐negative strains. *Staphylococcus aureus* recorded zones of inhibition ranging from 10 to 20 mm. *Enterococcus faecalis* had zones of inhibition from 8 mm for grape juice concentrate to 26 mm for tangerine juice concentrate. *Salmonella* spp. had the lowest zones of inhibition for the juices studied (6–10 mm). Tangerine produced the higher zones of inhibition for the bacterial strains studied while grape had the lowest zones of inhibition.

The lower zones of inhibition observed in the gram‐negative organisms compared to the gram‐positive organisms is not all together surprising. This is very likely due to the peptidoglycan‐containing periplasmic space and the outer membrane lipopolysaccharide layer of gram‐negative bacteria.

The gram‐negative outer membrane acts as a barrier, preventing the penetration of numerous environmental substances, including antimicrobial substances into the organism. The periplasmic space is also known to contain enzymes capable of breaking down foreign molecules attempting to gain entry into the microorganism (Holetz et al. 2002; Cheruiyot et al. 2009).

The antifungal activities of citrus juice concentrates revealed that *C. albicans* was most susceptible to the juice concentrates with observed zones of inhibition ranging from 8 mm for grape juice to 24 mm for lemon juice (Table 5), while *A. niger* and *Penicillum* spp. were less susceptible to the juice concentrates. Zones of inhibition ranging from 2 mm in tangerine juice to 6 mm for grape juice concentrates were observed for *A. niger*. However, *Penicillum* spp. was not susceptible to grape juice. While a 10‐mm zone of inhibition of *Penicillum* spp. growth was observed for lemon juice concentrates.

**Table 5:** Antifungal activities of some citrus juice concentrates (200 *μ*g/mL) against some fungal strains tested by disc diffusion assay

	Zone of inhibition (mm)		
	*Candida albicans*	*Aspergillus niger*	*Penicillum* spp.
Tangerine	14	2	4
Grape	8	6	–
Lemon	24	4	10
Lime	16	4	2

These results suggest that citrus juice concentrates at the concentration studied may not possess very good antifungal activities for controlling *A. niger* and *Penicillum* spp. growth as evidenced by the small zones of inhibition observed for these fungal strains. *Candida albicans* growth was however significantly inhibited by the juice concentrates.

This is especially important in light of the increasing incidence of candiadiasis infections as well as the emergence of resistant Candida species to existing antifungal drugs (Bardaweel et al. 2014).

Table 6 shows the inhibitory and bacteriocidal concentrations of the citrus juice concentrates against some bacterial strains. MIC values ranged from 12.25 to 100 *μ*g/mL, while MBC values ranged from 50 to 200 *μ*g/mL for all the juices studied. The results obtained showed that the MIC values for the juice concentrates were lower than their MBC values. Thus, suggesting that the concentrates were bacteriostatic at lower concentrations, but bacteriocidal at higher concentrations.

**Table 6:** Minimum inhibitory and bacteriostatic concentrations of some citrus juice concentrates against some bacterial strains expressed in *μ*g/mL

	Gram positive				Gram negative					
	*Staphylococcus aureus*		*Enterococcus faecalis*		*Pseudomonas aeruginosa*		*Escherichia coli*		*Salmonella* spp.	
	MIC	MBC	MIC	MBC	MIC	MBC	MIC	MBC	MIC	MBC
Tangerine	100	200	100	200	12.5	25	100	200	100	200
Grape	25	50	25	50	100	200	100	200	50	100
Lemon	25	50	50	100	12.5	50	50	100	25	50
Lime	12.5	50	50	100	25	50	25	50	12.5	50

MIC, minimum inhibitory concentration; MBC, minimum bacteriocidal concentration.

The grape, lemon, and lime juices were more effective in inhibiting the growth of *S. aureus*,* E. faecalis*, and *Salmonella* spp. Tangerine juice was most effective against *P. aeruginosa*.

Infections caused by *P. aeruginosa* are among the most difficult to treat with conventional antibiotics (Abu‐Shanab et al. 2004). Our study shows that tangerine and lemon juice concentrates significantly inhibited the growth of *P. aeruginosa* (MIC 12.5 *μ*g/mL). Lime juice concentrate also shows good inhibitory effect on the organism (MIC 25 *μ*g/mL). These juice concentrates may therefore hold promise in the management of *P. aeruginosa* infection.

*Staphylococcus aureus* is one of the most common bacteria implicated in food poisoning. The grape, lemon, and lime juice concentrates showed good inhibitory and bacteriocidal activities against this pathogen (MIC values of 25, 25, and 12.5 *μ*g/mL, respectively).

*Escherichia coli* was not as susceptible to the juice concentrates. Higher MIC values of the extracts were observed (25 *μ*g/mL for lime juice, 50 *μ*g/mL for lemon juice, and 100 *μ*g/mL for tangerine and grape juice). *Escherichia coli* is a naturally occurring bacteria in the intestinal tract of man. The acquisition of invasion factors increases their ability to adapt to new niches and their disease‐causing abilities (Akinjogunla et al. 2010). Our results show that the lime juice concentrate may be an alternative source of antimicrobial agent for this organism.

The inhibitory and fungicidal concentrations of the citrus juice concentrates against three fungal strains is shown in Table 7. The MFC was observed to be higher than the MIC in all the juices. *Candida albicans* was the most susceptible organism with MIC values of 25 *μ*g/mL for tangerine, grape, and lemon juices. *Penicillum* spp. was least susceptible to the juice concentrates with MIC values of 100 *μ*g/mL for lemon and lime juices and 50 *μ*g/mL for tangerine juice. *Penicillum* spp. was not susceptible to grape juice.

**Table 7:** Minimum inhibitory and fungicidal concentrations of some citrus juice concentrates against some fungal strains expressed in *μ*g/mL

	*Candida albicans*		*Aspergillus niger*		*Penicillum* spp.	
	MIC	MFC	MIC	MFC	MIC	MFC
Tangerine	25	50	100	200	50	100
Grape	25	50	100	200	–	–
Lemon	25	50	50	100	100	200
Lime	50	100	50	100	100	200

MIC, minimum inhibitory concentration; MFC, minimum fungicidal concentration.

The results of this study showed that grape juice was least effective both as an antibacterial and as an antifungal agent. This is evidenced by the smaller zones of inhibition and larger MIC, MBC, and MFC values obtained for grape juice concentrate. However, grape juice had more promising antioxidant activities than the other juice concentrates as evidenced by its higher FRAP values and high percentage of inhibition of the DPPH radical. This juice may therefore have other health benefits that may not necessarily be antimicrobial.

A lot of data exists on the antimicrobial activities of a wide range of extracts of plant origin. This is especially useful for dwellers in rural communities in developing countries who have limited access to synthetic antimicrobial drugs. This study has therefore provided additional information on the health benefits of consuming fruits.

The results of this study have revealed that these commonly consumed citrus juices may contain promising antimicrobial leads. This study, however, provides in vitro data which may not be exactly replicated in vivo. Further studies directed at isolation of novel antimicrobial compounds and in vivo studies that may validate the in vitro findings are recommended.

## Conflict of Interest

None declared.
